# Editorial: Intraoperative Radiotherapy (IORT)—A New Frontier for Personalized Medicine as Adjuvant Treatment and Treatment of Locally Recurrent Advanced Malignancy

**DOI:** 10.3389/fonc.2018.00234

**Published:** 2018-06-25

**Authors:** Tarita O. Thomas, William Small

**Affiliations:** Loyola University Medical Center, Maywood, IL, United States

**Keywords:** intraoperative radiotherapy, personalized medicine, Kv, breast cancer, physics

**Editorial on the Research Topic**

**Editorial: Intraoperative Radiotherapy (IORT)—A New Frontier for Personalized Medicine as Adjuvant Treatment and Treatment of Locally Recurrent Advanced Malignancy**

Intraoperative radiotherapy (IORT) is a treatment delivery technique with reports starting in the early twentieth century with the use of orthovoltage energy with limited applicability due to the energy characteristics (1). This technology had a resurgence in approximately the 1960s with the use of electron energy in Japan (2) and subsequently the literature is replete with numerous other publications. Initially, the use of IORT was restricted by the cumbersome nature of treatment delivery as a shielded operating room was needed requiring large capital expenditure as well as expertise of staff with limited patient applicability. Over time the technology has evolved from requiring a shielded room to the development of a mobile device that can move into a standard operating room with minimal shielding requirements. Various current technologies exist that deliver electron or photon energies intraoperatively including Novac7 (Hitesys SPA, Aprillia, Italy; 7–10 MeV), Mobetron (IntraOp Medical Corporation, Sunnyvale, CA, USA; 4–12 MeV), Axxent system (Xoft, San Jose, CA, USA; 20–50 kV), and Intrabeam system (Carl Zeiss Meditec, Dublin, CA, USA; 30–50 kV).

There are numerous advantages of IORT in oncology. IORT has the benefit of delivering a tumoricidal radiation dose in a single treatment, while targeting the therapy to the region of highest risk of disease recurrence with direct visualization in the operating room. This provides a high relative biological effectiveness while limiting dose to normal tissue *via* tumor bed devascularization, elimination of inter-fraction tumor cell repopulation, and possibly providing a systemic immune effect (3). In addition, there are practical benefits to the patient by elimination or reduction in outpatient treatment visits that routinely last for 5–6 weeks for conventional postoperative radiotherapy, such as improved quality of life, decreased side effects and financial advantages.

In this series we focus on the use, radiobiology, and physics of IORT with an emphasis on the Intrabeam system. Sethi et al. describe the technical and dosimetric considerations for the various applicators now available to treat patients with disease intraoperatively in various locations including with flat, spherical, and even a needle applicator. Valente et al. discuss their experience with IORT from the surgical perspective and how their group decreased operative times in patients receiving breast IORT with increased utilization. Paunesku and Woloschak provide a review of the history of IORT as well as an engaging discussion of how IORT can be used in the future. Herskind et al. extend this discussion into the theoretical usage of large radiation fraction size in brain metastasis and the potential combination with immunotherapy. The series then reviews the use of IORT in various malignancies, including head and neck cancer, pancreas cancer, and brain metastasis. Three articles finally review the use of IORT in breast cancer a highly prevalent cancer with numerous radiation treatment options available. Jacobson and Sochi provide a review of the various types of partial breast therapy and the toxicities associated. Chin et al. describe their experience using IORT for patients with prior thoracic radiation exposure. Harris and Small provide a comprehensive review of the data in support of the use of breast IORT as well as the toxicities, cosmesis, and quality of life with use of this treatment modality touching on both the use of electron and photon-based IORT.

The Organization for Economic Cooperation and Development evaluated the spending, supply, utilization, and price of health care across 13 high income countries and found that as a percent of GDP from 1980 to 2013 health care spending is approximately 17% in the United States versus 10% in the other countries evaluated (4). In the United States, health care spending consumes on average 1/5 of a households’ income. It has been estimated that the use of breast IORT could provide at least $1.2 billion in saving to the health care system in the United States over 5 years (5). Currently breast IORT using the IntraBeam system is being used in 35 countries in more than 300 major hospitals with more than 200,000 women treated. Here, we provide a series of articles that discuss the usage of IORT in various malignancies as well as the technical aspects of this technology. As medicine and health continue to evolve the new frontier of personalized medicine must continue to rigorously evaluate and implement technologies that limit costs to the health care system and provide meaningful therapeutic benefit.

## Author Contributions

Both authors contributed to the editorial.

## Conflict of Interest Statement

The authors declare that the research was conducted in the absence of any commercial or financial relationships that could be construed as a potential conflict of interest.
